# Non-Essential Role for TLR2 and Its Signaling Adaptor Mal/TIRAP in Preserving Normal Lung Architecture in Mice

**DOI:** 10.1371/journal.pone.0078095

**Published:** 2013-10-29

**Authors:** Saleela M. Ruwanpura, Louise McLeod, Andrew R. Lilja, Gavin Brooks, Lovisa F. Dousha, Huei J. Seow, Steven Bozinovski, Ross Vlahos, Paul J. Hertzog, Gary P. Anderson, Brendan J. Jenkins

**Affiliations:** 1 Centre for Innate Immunity and Infectious Diseases, Monash Institute of Medical Research, Monash University, Clayton, Victoria, Australia; 2 Department of Medicine and Pharmacology, The University of Melbourne, Parkville, Victoria, Australia; University of Pittsburgh, United States of America

## Abstract

Myeloid differentiation factor 88 (MyD88) and MyD88-adaptor like (Mal)/Toll-interleukin 1 receptor domain containing adaptor protein (TIRAP) play a critical role in transducing signals downstream of the Toll-like receptor (TLR) family. While genetic ablation of the TLR4/MyD88 signaling axis in mice leads to pulmonary cell death and oxidative stress culminating in emphysema, the involvement of Mal, as well as TLR2 which like TLR4 also signals via MyD88 and Mal, in the pathogenesis of emphysema has not been studied. By employing an *in vivo* genetic approach, we reveal here that unlike the spontaneous pulmonary emphysema which developed in *Tlr4^−/−^* mice by 6 months of age, the lungs of *Tlr2^−/−^* mice showed no physiological or morphological signs of emphysema. A more detailed comparative analysis of the lungs from these mice confirmed that elevated oxidative protein carbonylation levels and increased numbers of alveolar cell apoptosis were only detected in *Tlr4^−/−^* mice, along with up-regulation of NADPH oxidase 3 (*Nox3*) mRNA expression. With respect to Mal, the architecture of the lungs of *Mal^−/−^* mice was normal. However, despite normal oxidative protein carbonylation levels in the lungs of emphysema-free *Mal^−/−^* mice, these mice displayed increased levels of apoptosis comparable to those observed in emphysematous *Tlr4^−/−^* mice. In conclusion, our data provide *in vivo* evidence for the non-essential role for TLR2, unlike the related TLR4, in maintaining the normal architecture of the lung. In addition, we reveal that Mal differentially facilitates the anti-apoptotic, but not oxidant suppressive, activities of TLR4 in the lung, both of which appear to be essential for TLR4 to prevent the onset of emphysema.

## Introduction

Emphysema is a major component of the debilitating chronic obstructive pulmonary disease (COPD), the fifth most common cause of death world-wide [1]. While cigarette smoke (CS) exposure is the primary risk factor for COPD, only 10–20% of heavy smokers develop clinically-significant emphysema, and approximately 20% of patients with emphysema are non-smokers [2]. Collectively, these observations suggest that factors intrinsic to the host (i.e. of genetic origin) are likely to determine an individual’s susceptibility to emphysema.

Over the last decade, it has become apparent that the interplay between oxidant/antioxidant imbalances, elevated apoptosis, excessive protease activity and chronic inflammation are pivotal to the pathogenesis of emphysema [3]–[6]. Since CS is a rich source of oxidants, the molecular mechanisms regulating the balance between oxidant and anti-oxidant production in the lung have long been thought to be critical in promoting emphysema. In this regard, an association between the regulation of oxidative stress by the innate immune system, and its role in emphysema, has recently emerged. Specifically, a causal role for TLR4, the prototypical innate immune pathogen recognition receptor, in the development of emphysema has been suggested from animal studies whereby TLR4 deficiency leads to spontaneous emphysema as a result of excessive oxidant activity [3]. Furthermore, clinical studies have demonstrated TLR4 polymorphisms and down-regulated TLR4 expression in lung tissues from emphysematous smokers [7], [8]. Despite these observations, the downstream signaling pathway(s) by which TLR4 regulates pulmonary oxidative stress levels and protects against emphysematous changes in the lung are not fully understood.

Among the TLR family, TLR2 recognizes the broadest spectrum of pathogen-associated molecular patterns, including bacterial peptidoglycans and lipoproteins, whereas TLR4 mediates responses primarily to lipopolysaccharide from Gram negative bacteria [9]. All TLRs have a conserved intracytoplasmic Toll/interleukin (IL)-1 receptor (TIR) domain which is responsible for signal transduction. The TIR-domain containing MyD88 adaptor is essential for signaling by all TLR (and IL-1R) family members, with the exception of TLR3 [10]. Another adaptor protein closely related to MyD88 is the TIR domain-containing Mal/TIRAP [11], which together with MyD88 is essential for the activation of nuclear factor kappa beta (NF-κB), and p38, extracellular-regulated kinase (ERK) and c-Jun N-terminal kinase (JNK) mitogen-activated protein kinase (MAPK) pathways via TLR4 and the related TLR2 [11], [12]. Interestingly, both TLR4 and TLR2 mediated signaling also leads to the rapid activation of the phosphoinositide-3-kinase (PI3K)/Akt pathway, which is involved in the regulation of apoptosis and cell growth [13]–[15]. Although TLR4 deficiency causes an oxidative imbalance in the lung leading to emphysema in a MyD88-dependent manner [3], the role of Mal in emphysema has not been studied. In addition, while TLR2 has been associated with enhancing airway inflammation and immunity by facilitating the production of cytokines in COPD [16]–[18], the involvement of TLR2 in emphysema development is not known.

Here we report a differential requirement for TLR2 and TLR4 in protecting against apoptosis and regulating the oxidative balance in the lung, since *Tlr2^−/−^* mice display normal pulmonary oxidative stress and apoptosis levels, and also fail to develop emphysema. By contrast, the spontaneous development of emphysema in *Tlr4^−/−^* mice strongly correlated with elevated oxidative protein carbonylation levels and increased alveolar cell apoptosis by 6 months of age. Strikingly, although Mal deficiency in the lungs of mice promoted increased alveolar cell apoptosis comparable to that observed upon TLR4 deficiency, oxidative stress levels in *Mal^−/−^* mice were normal, and *Mal^−/−^* mice did not develop emphysema. Taken together, our findings provide *in vivo* evidence for the non-obligate requirement for Mal (unlike MyD88) specifically by TLR4 in the lung. Furthermore, we propose that both excessive pulmonary apoptosis and oxidative stress caused by the absence of TLR4 are required for the pathogenesis of emphysema.

## Materials and Methods

### Mice

Mice homozygous null for *Tlr4* (*Tlr4^−/−^*) [19] and *Tlr2* (*Tlr2^−/−^*) [20] on a C57BL6 background were used with their genetically-matched wild-type controls. Mice homozygous null for the *Mal* gene (*Mal^−/−^*) [21] on 129/C57BL6 background were used with matching background wild-type control mice. Experiments involving cigarette smoke exposure of mice over 4 days were performed as described previously [6]. All experiments were fully approved by the Monash Medical Centre “A” and University of Melbourne Animal Ethics Committees, and all animals were housed under specific pathogen-free conditions.

### Mouse Tissue Collection

The collection of mouse lungs for stereology, histology and immunohistochemistry was performed as previously described [6],[22].

### Human Lung Samples

Lung tissue from resection surgery for treatment of a solitary peripheral carcinoma was collected from patients with either no evidence of emphysema or moderate emphysema, as defined by pulmonary function tests (gas exchange, FEV1) and histopathology (Table 1). Lung tissue was collected from individuals, upon formal written informed consent, with either no evidence of emphysema (n = 9) or moderate emphysema (n = 14), and then snap frozen in liquid nitrogen. Studies were approved by the Southern Health Human Research Ethics Committee.

**Table 1:** Clinical characteristics of patients.

Characteristics	Sub-characteristics	No emphysema (n = 9)	Moderate emphysema (n = 14)
**Sex (patient number)**	Male	5	8
	Female	4	6
**Smoking history** **(Pack years)**		53±30	52±15
**Age (years)**		69±5	71±2
**Diagnosis**		Lung cancer (tissues were obtained from non-cancer area)	Lung cancer (tissues were obtained from non-cancer area)
**FEV1 (% of predicted post)-COPD status**		93.8±6.7	79.4±4.5
**DLCO (gas transfer, % predicted** **post)-emphysema status**		75.1±4.5	51.1±2.0

Data are expressed as the mean ± SEM. FEV1, forced expiratory volume in one second. DLCO, diffusing capacity of the lungs for carbon monoxide.

### Stereological Analyses

Lung stereology was performed on methylene blue-stained tissue sections using computer-assisted newCAST software (version 2.14; Visiopharm, Hørsholm, Denmark) [6], [22].

### Lung Function Analyses

The assessment of lung function on anesthetized mice was performed using the flexiVent system (SCIREQ, Montreal, Canada) [6], [22].

### Immunohistochemistry and Immunofluorescence

Immunohistochemistry for the detection of apoptosis was determined by the TUNEL technique using an ApopTag Peroxidase In Situ Apoptosis Detection kit (Millipore, Billerica, MA). Stereological techniques were applied to determine the number of alveolar septal TUNEL-stained cells per 20 fields [22].

### Protein Extraction and Immunoblotting

Total protein lysates were prepared from snap-frozen lung tissue and subjected to immunoblotting for Actin [6], [22]. Proteins were visualized using the Odyssey Infrared Imaging System (LI-COR, Lincoln, NE) and quantified using the Image J program (nih.gov).

### Oxyblot Assay

Immunoblotting was performed to identify dinitrophenylhydrazone derivatives of protein carbonyls using the Protein Oxidation Kit (Millipore, Billerica, MA). Proteins were visualized by autoradiography, and the intensity of each lane was quantified by densitometric scanning and normalized against actin protein levels.

### RNA Isolation and Gene Expression Analysis

Total RNA extraction and the preparation of cDNA for quantitative RT-PCR (Q-PCR) expression analyses of individual genes were performed as previously described [6], [22]. Sequences for mouse primers are as follows; *Nox2* forward 5′-CTTATCACAGCCACAAGCATT-3′, *Nox2* reverse 5′-CACCCATTCACACTGACCTCT-3′; *Nox3* forward 5′-GCTTGTGGCTGTGATAAGCA-3′, *Nox3* reverse 5′-CACTGGCTGTACCAAAGGGT-3′. Sequences for human primers are as follows; *TLR1* forward 5′-CAGTGTCTGGTACACGCATGGT-3′, *TLR1* reverse 5′- TTTCAAAAACCGTGTCTGTTAAGAGA-3′; *TLR2* forward 5′-GCCTCTCCAAGGAAGAATCC-3′, *TLR2* reverse 5′-TCCTGTTGTTGGACAGGTCA-3′; *TLR3* forward 5′-CCTGGTTTGTTAATTGGATTAACGA-3′, *TLR3* reverse 5′-TGAGGTGGAGTGTTGCAAAGG-3′; *TLR4* forward 5′-AAGCCGAAAGGTGATTGTTG-3′, *TLR4* reverse 5′-CTGAGCAGGGTCTTCTCCAC-3′; *TLR5* forward 5′- TGCCTTGAAGCCTTCAGTTATG-3′, *TLR5* reverse 5′-CCAACCACCACCATGATGAG-3′; *TLR6* forward 5′-GAAGAAGAACAACCCTTTAGGATAGC-3′, *TLR6* reverse 5′-AGGCAAACAAAATGGAAGCTT-3′; *MAL* forward 5′-TCACGGGGCTAGTTTTGACC-3′, *MAL* reverse 5′-TCTTGGGCTTCTTCAGCAGG-3′; *18S* forward 5′-CGGCTACCACATCCAAGGAA-3′, *18S* reverse 5′-GCTGGAATTACCGCGGCT-3′.

### Statistical Analyses

All statistical analyses were performed using GraphPad Prism for Windows version 5.0. As data were normally distributed, one-way ANOVA or student t-test was used to determine differences between genotypes. A *P*<0.05 was considered statistically significant. Data are expressed as the mean ± SEM.

## Results

### Genetic Ablation of TLR4, but not TLR2, Leads to Pulmonary Emphysema in Mice

**Figure 1:**
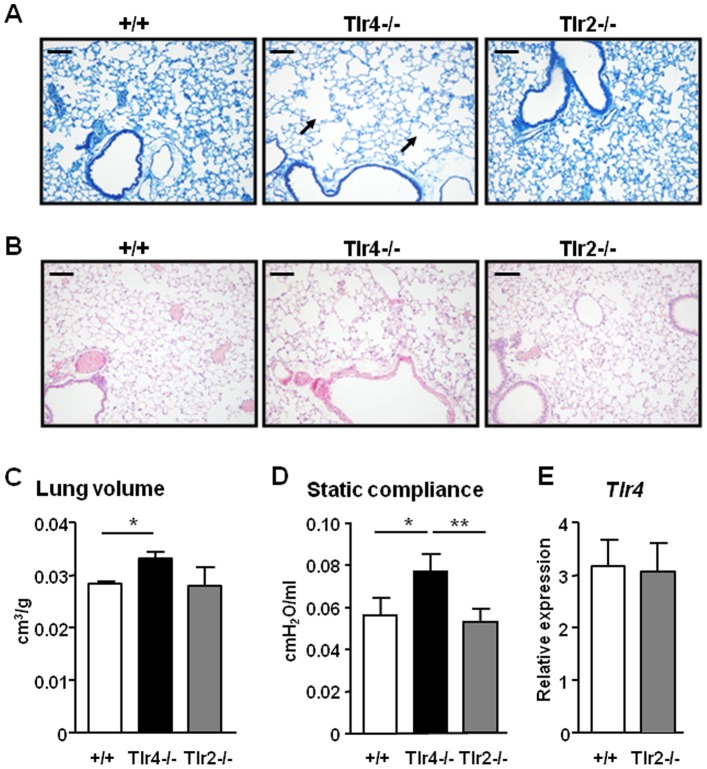
Alveolar air space enlargement, increased lung volume and static compliance in *Tlr4^−/−^*, but not *Tlr2^−/−^*, mice. (**A**) Representative methylene blue stained cross-sections and (**B**) representative H&E stained cross-sections of lungs from wild-type (+/+), *Tlr4^−/−^* and *Tlr2^−/−^* mice aged 6 months. Arrows indicate enlarged airspaces. Scale bars = 100 µm (**C**) Lung volume per body weight of +/+ and *Tlr4^−/−^* and *Tlr2^−/−^* mice. (**D**) Static compliance of 6 month old +/+, *Tlr4^−/−^* and *Tlr2^−/−^* mice was assessed by flexiVent lung function analysis. Data are expressed as mean ± SEM, n = at least 3 mice per genotype. *P<0.05, **P<0.01 versus age-matched +/+ mice. (**E**) Q-PCR expression analyses of *Tlr4* was performed on lung cDNA from 6 month old +/+ and *Tlr2^−/−^* mice. Expression data are shown from n = 4 per genotype following normalization for *18S* expression, and are presented as mean ± SEM from triplicate analysis.

Since emphysema is caused by *Tlr4* gene disruption in mice [3], [23], and emphysema in smokers correlates with impaired *TLR4* expression [7], [8], we initially investigated the specificity of the requirement for TLR4 to protect against emphysema by comparing the lung phenotypes of mice deficient in *Tlr4* and the closely-related *Tlr2* gene. Consistent with a previous study [3], histological evaluation of lung sections from unchallenged *Tlr4^−/−^* mice at 6 months of age revealed emphysema as characterized by enlargement of the distal air spaces and destruction of normal alveolar architecture (Figure 1A) without any obvious inflammation (Figure 1B). In addition, the lung volume of *Tlr4^−/−^* mice was significantly increased compared to age matched wild-type control mice (Figure 1C). By contrast, the histological evaluations and lung volume measurements of 6 month old *Tlr2^−/−^* mice revealed no airspace enlargement or pulmonary inflammation, and lung volumes were comparable to wild-type control mice (Figure 1A–C).

Stereological analyses were next employed to further define changes in lung morphology associated with emphysematous alterations [6], [22], [24]. The *Tlr4^−/−^* mice showed significant increases in the volume fractions of airspace within parenchyma tissue, as well as per lung, at 6 months of age (Table 2). These findings are consistent with significant reductions in the volume fraction and volume of alveolar septal tissue within lung parenchyma of *Tlr4^−/−^* mice at 6 months of age (Table 2), which reflect a loss of alveolar tissue in lung parenchyma as a result of alveoli destruction, a hallmark of emphysema. For *Tlr2^−/−^* mice, however, all of the above stereological parameters remained unchanged and were similar to control wild-type littermates (Table 2).

**Table 2:** Comparative stereological analyses of lungs from wild-type (+/+), Tlr4^−/−^ and Tlr2^−/−^ mutant mice.

	*+/+*	*Tlr4^−/−^*	*Tlr2^−/−^*
**Vv (par/lung) (%)**	82.3 (±0.7)	88.0 (±0.3)**	80.9 (±1.2)
**Vv (airsp/par) (%)**	65.5 (±1.1)	78.1 (±1.0)***	63.9 (±0.7)
**V (airsp/lung) (cm^3^)**	48.8 (±2.0)	69.2 (±1.8)***	44.9 (±3.0)
**Vv (sep/par) (%)**	26.7 (±0.8)	18.4 (±0.2)***	25.4 (±0.8)
**V (sep/lung) (cm^3^)**	19.9 (±0.7)	16.3 (±0.5)*	17.8 (±0.8)
**Sv (sep/par) (1/cm)**	667 (±7.5)	628 (±11)	675 (±36)
**S (sep/lung) (cm^2^)**	528 (±5.9)	553 (±10)	540 (±6.3)

Data are expressed as the mean ± SEM. n = at least 5 mice per genotype. *P<0.05, **P<0.01 and ***P<0.001 versus age-matched +/+ mice. Vv = volume fraction; par = parenchyma; air = air space; sep = septal tissue; Sv = surface density; S = surface area.

To validate the functional relevance of the histology and stereology data demonstrating emphysematous changes in the lungs of *Tlr4^−/−^* mice, but not *Tlr2^−/−^* mice, we next assessed lung static compliance of mice using the forced oscillatory technique [6], [22], [25]. As shown in Figure 1D, the static compliance was significantly increased in *Tlr4^−/−^* mice compared to wild-type mice, while it remained unchanged in *Tlr2^−/−^* mice at 6 months of age. In addition, the mRNA levels of *Tlr4* were comparable in the lungs of wild-type and *Tlr2^−/−^* mice (Figure 1E), thus confirming that the genetic ablation of *Tlr2* had no compensatory effect on augmenting *Tlr4* gene expression in the lung which might potentially mask detrimental effects of TLR2-deficiency.

**Figure 2:**
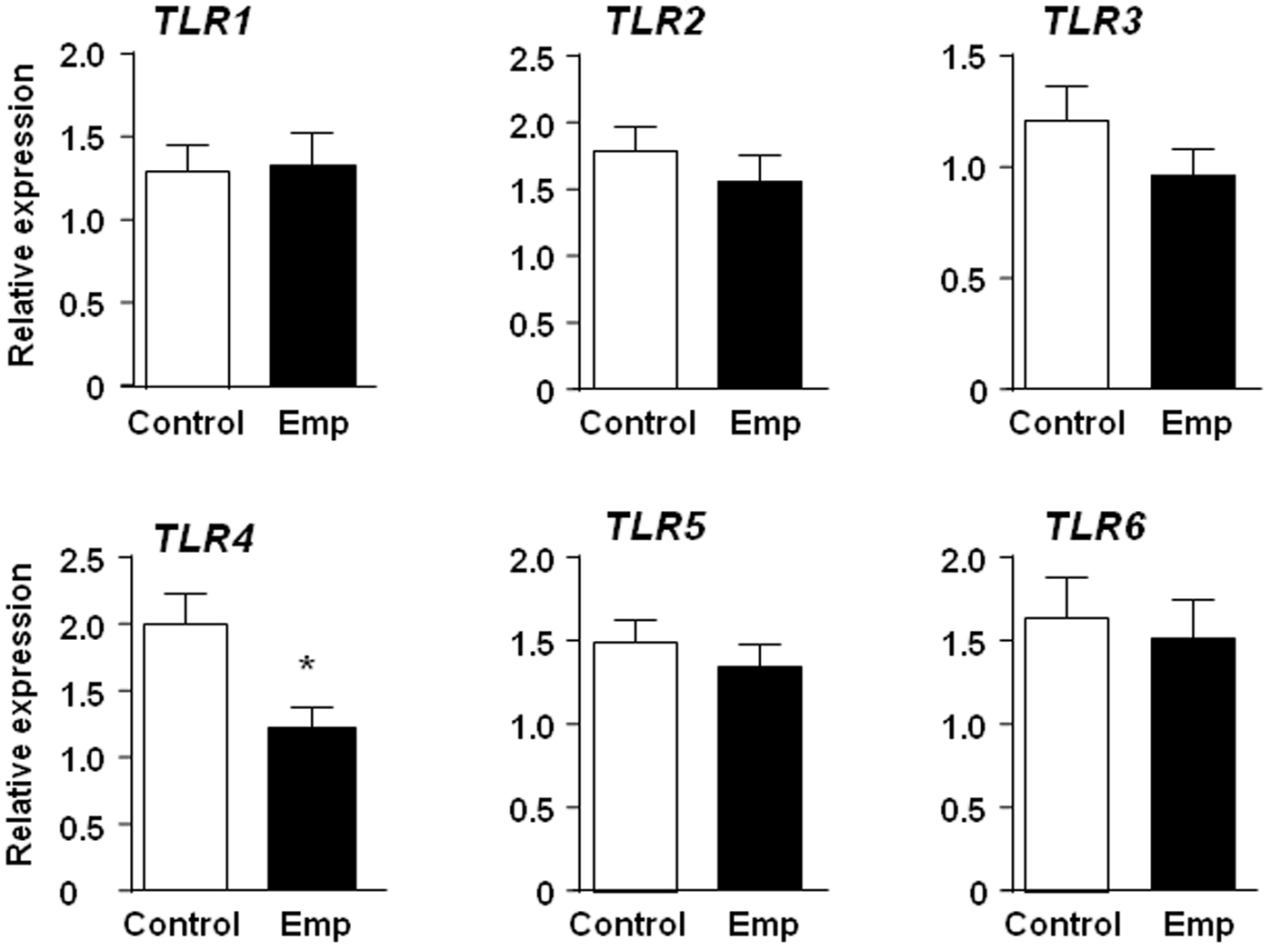
Reduced gene expression of *TLR4*, but not other TLR family members, in human emphysema tissue. Q-PCR gene expression analyses of *TLRs 1–6* were performed on human lung cDNA from emphysema (Emp) patients (n = 14) or emphysema-free (Control) individuals (n = 9). Expression data are shown following normalization for *18S* expression, and are presented from triplicate analysis as the mean ± SEM. *P<0.05 versus emphysema-free controls.

Furthermore, these observations that the specific reduction of *Tlr4*, but not *Tlr2*, gene expression in the mouse correlated with the onset of emphysema were also validated in human disease, whereby among members of the TLR family, only *TLR4* gene expression was significantly suppressed in human lung tissue from individuals with emphysema versus emphysema-free controls (Figure 2). Collectively, these data demonstrate a non-essential role of TLR2 in emphysema development.

### Increased Apoptosis and Oxidative Stress are Emphysematous Processes Specific to the Lungs of *Tlr4^−/−^*, but not *Tlr2^−/−^,* Mice

**Figure 3:**
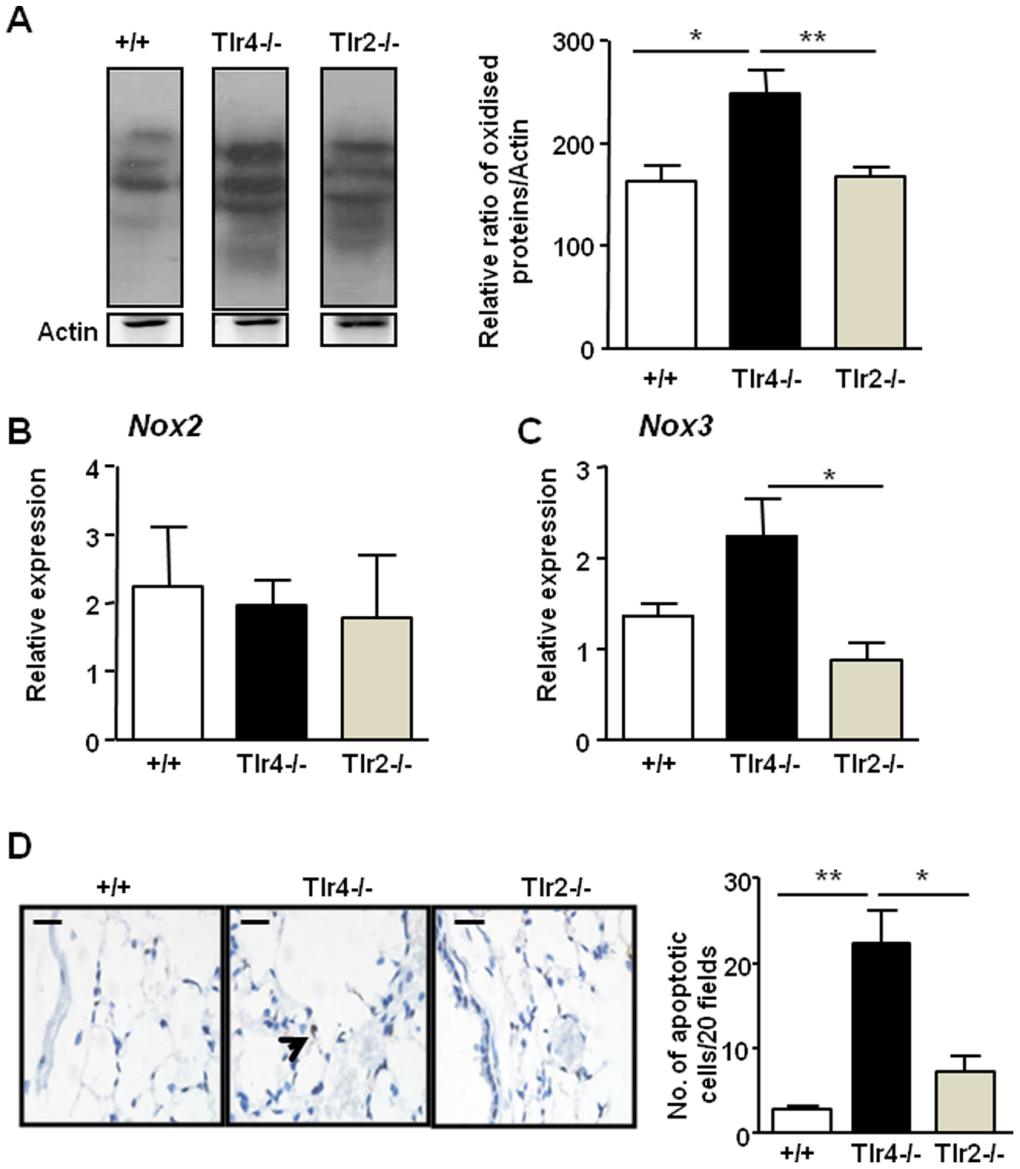
Increased oxidative stress and pulmonary apoptosis in *Tlr4^−/−^*, but not *Tlr2^−/−^*, mice. (**A**) Protein carbonylation levels were determined by Oxyblot of lung protein lysates from 6 month old +/+, *Tlr4^−/−^* and *Tlr2^−/−^* mice. Each lane represents tissue from an individual mouse. Densitometric quantification of total protein carbonylation in representative samples per genotype was performed and normalized against actin protein levels present in each sample. Data are presented as the mean fold induction ± SEM for at least n = 4 per genotype. Q-PCR expression analyses of (**B**) *Nox2* and (**C**) *Nox3* were performed on lung cDNA from 6 month old mice of the indicated genotypes. Expression data are shown from at least n = 4 per genotype following normalization for *18S* expression, and are presented from triplicate analysis. (**D**) Representative photomicrographs showing TUNEL-stained cells in a cross-section of the lungs from the indicated genotypes at 6 months of age. Stereological quantification of the percentage of TUNEL-stained cells in the lungs of mice. Data are expressed as mean ± SEM, n = at least 3 mice per genotype. *P<0.05, **P<0.01 versus age-matched +/+ mice.

Oxidative imbalance is a key process associated with the development of emphysema in both COPD patients and mouse disease models, including mice lacking TLR4 [3], [26]. In contrast, there is evidence for a role for TLR2 in activating oxidative stress in COPD [27], [28] and airway inflammation [29]. We therefore next determined whether the differential role of TLR4 and TLR2 in protecting against the onset of emphysema correlated with alterations in the oxidant/antioxidant ratio in *Tlr4^−/−^* and *Tlr2^−/−^* mice. Immunoblotting for dinitrophenylhydrazone derivatives of protein carbonyls, a conventional oxidative stress marker generated by reactive oxygen species, confirmed that there were no qualitative or quantitative differences in protein carbonyl formation in the lungs of *Tlr2^−/−^* mice. By contrast, increased oxidative stress was observed in the lungs of *Tlr4^−/−^* mice compared to their wild-type controls (Figure 3A). Previously, intracellular oxidants such as those derived from the NADPH oxidase (Nox) system have been implicated in disease states [30], [31], and TLR4 deficiency in mice led to the up-regulation of Nox3 in lungs which correlated with the emphysema phenotype [3]. Therefore, we also examined mRNA expression levels of *Nox2*, the most commonly described catalytic component of the Nox system, and *Nox3* in the lungs of *Tlr4^−/−^* and *Tlr2^−/−^* mice. While *Nox2* mRNA expression levels remained unchanged for all genotypes (Figure 3B), *Nox3* was elevated only in the lungs *Tlr4^−/−^* mice (Figure 3C).

Another major cellular process associated with the development of human emphysema and animal models of emphysema, including TLR4-deficient mice, is apoptosis [3], [6], [22], [32], [33]. Similarly, it is found that TLR2 and TLR4 double knockout mice following the treatment of bleomycin, a non-infectious model of lung injury, led to increase level of lung cell apoptosis by suggesting the TLR2/4 together play a protective role in cell survival to prevent destruction of tissue integrity and allowing for tissue repair [34], [35]. However, it must be noted that in above animal models, the results regarding the individual roles of TLR2 and TLR4 in protecting lung apoptosis were not studied. We therefore next compared the levels of apoptosis in the lungs of *Tlr4^−/−^* and *Tlr2^−/−^*6 month old mice by performing TUNEL staining on lung sections. As shown in Figure 3D, we observed both qualitative and quantitative (by stereological analysis) elevation in the numbers of TUNEL-stained cells in lungs of *Tlr4^−/−^* mice with the predominant cell type affected being alveolar septal cells, while no apoptotic changes were detected in the lungs of *Tlr2^−/−^* mice compared to control mice. Collectively, the above data indicate that, unlike TLR4, neither oxidative stress nor apoptosis is affected by the loss of TLR2 signaling in the mouse lung.

### Differential Requirement for Mal in the TLR4-mediated Protection Against Pulmonary Cell Death and Oxidative Stress

**Figure 4:**
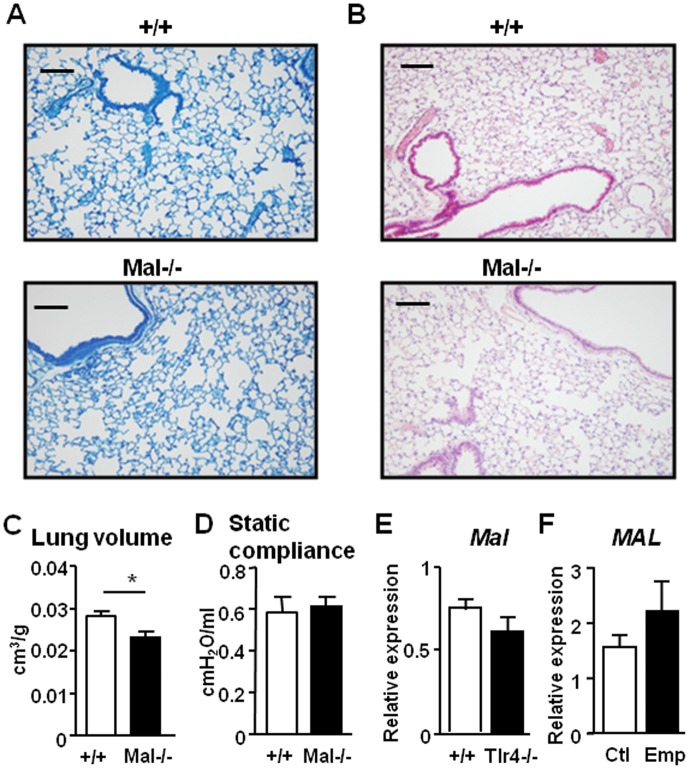
No changes in the emphysematous parameters in *Mal^−/−^* mice. (**A**) Representative methylene blue stained cross-sections and (**B**) representative H&E stained cross-sections of lungs from *Mal^+/+^* (+/+) and *Mal^−/−^* mice aged 6 months. Scale bars = 100 µm (**C**) Lung volume per body weight of +/+ and *Mal^−/−^* mice (**D**) Static compliance of 6 month old +/+ and *Mal^−/−^* mice was assessed by flexiVent lung function analysis. Data are expressed as mean ± SEM, n = at least 3 mice per genotype. *P<0.05 versus age-matched +/+ mice. (**E**) Q-PCR expression analyses of *Mal* was performed on lung cDNA from 6 month old +/+ and *Tlr4^−/−^* mice. Expression data are shown from n = 4 per genotype per following normalization for *18S* expression, and are presented as mean ± SEM from triplicate analysis. (**F**) Q-PCR expression analysis of *MAL* was performed on human lung cDNA from emphysema (Emp) patients (n = 14) or emphysema-free (Ctl) individuals (n = 9). Expression data are shown following normalization for *18S* expression, and are presented from triplicate analysis as the mean ± SEM.

Since Mal is a key downstream signaling adaptor of both TLR4 and TLR2 [11], the emphysematous and normal appearance of lungs from *Tlr4^−/−^* and *Tlr2^−/−^* mice, respectively, suggests that the pulmonary function(s) of Mal may preferentially align with either TLR2 or TLR4. Histological evaluation of *Mal^−/−^* mice revealed morphologically normal alveolar architecture (Figure 4A) with no apparent inflammation (Figure 4B) at 6 months of age. Furthermore, both the lung volume and static lung compliance of *Mal^−/−^* mice were comparable to those of wild-type control mice (Figure 4C and 4D). In support of these findings, stereological assessment of *Mal^−/−^* mouse lungs revealed that the volume fractions of air space and alveolar septal tissue, and surface density within lung parenchyma, as well as absolute volumes of lung, were normal at 6 months (Table S1). Notably, there was no significant change in the expression of *Mal* in the lungs of emphysematous *Tlr4* ^−/−^ mice (Figure 4E), and the lack of a role for Mal in the development of emphysema in human disease was also suggested by our observation that *MAL* gene expression was unaltered in human lung tissue from emphysema and control emphysema-free individuals (Figure 4F).

**Figure 5:**
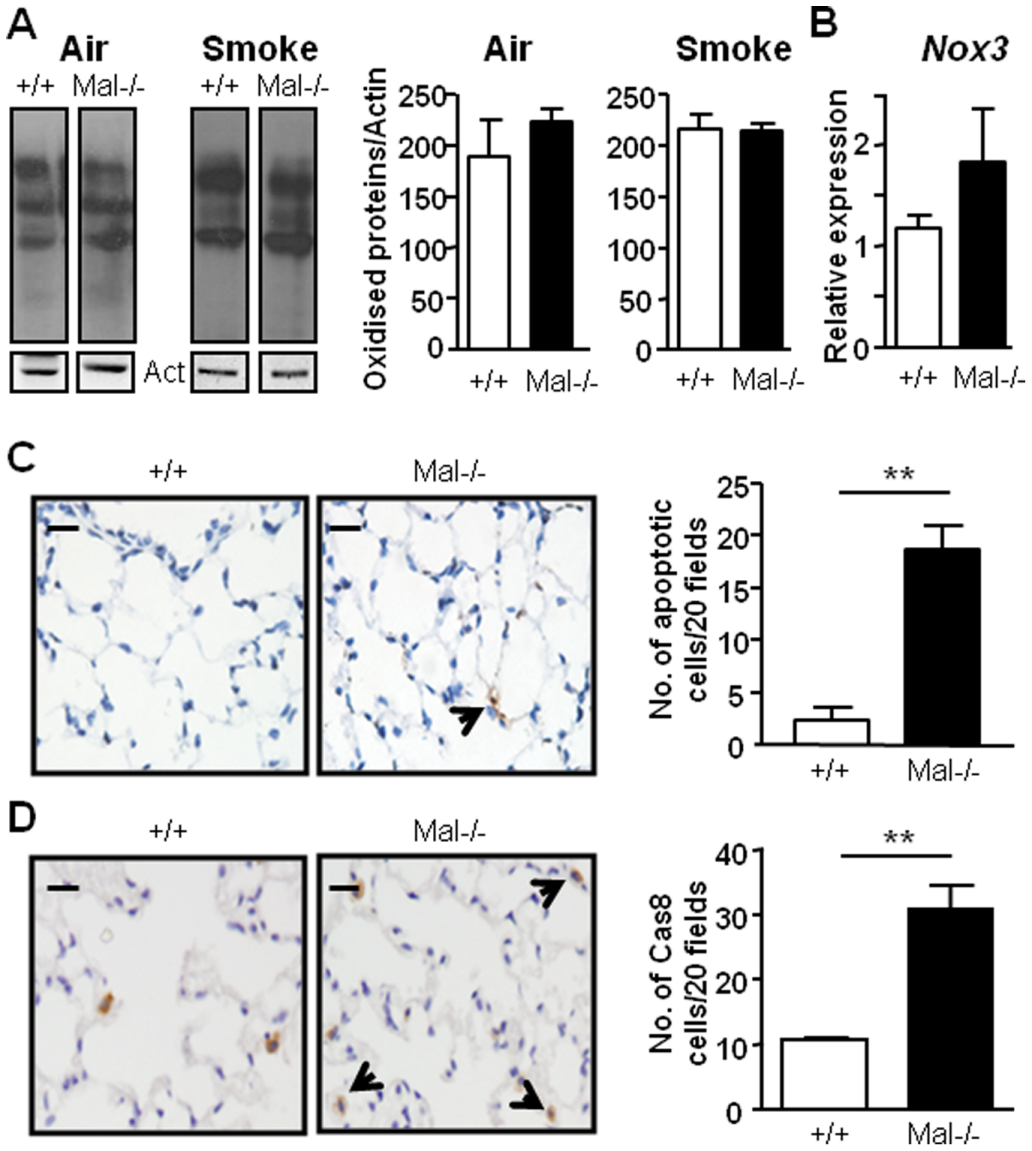
Altered lung cell apoptosis, but not oxidative stress, in *Mal^−/−^* mice. (**A**) Protein carbonylation levels were determined by Oxyblot of lung protein lysates from +/+ and *Mal^−/−^* mice that were either exposed to air or were exposed to cigarette smoke for 4 days. Each lane represents tissue from an individual mouse. Densitometric quantification of total protein carbonylation in representative samples per genotype was performed and normalized against actin protein levels present in each sample. Data are presented as the mean fold induction ± SEM for at least n = 4 per genotype. (**B**) Q-PCR expression analyses of *Nox3* was performed on lung cDNA from 6 month old +/+ and *Mal^−/−^* mice. Expression data are shown from n = 4 per genotype following normalization for *18S* expression, and are presented as mean ± SEM from triplicate analysis. (**C**) Representative photomicrographs showing TUNEL-stained cells and (**D**) active-caspase 8 (Cas8)-stained cells in a cross-section of the lungs from the indicated genotypes at 6 months of age. Graphs depict stereological quantification of the percentage of TUNEL and Cas8-stained cells in the lungs of mice. Data are expressed as mean ± SEM, n = at least 3 mice per genotype. **P<0.01, versus age-matched +/+ mice.

In light of the absence of an emphysema phenotype in the lungs of *Mal^−/−^* mice, unlike the emphysema observed in *Tlr4^−/−^* mice, we next assessed whether the levels of oxidative stress and apoptosis (both elevated in *Tlr4^−/−^* lungs) were also unaltered in *Mal^−/−^* lungs. Indeed, the lung lysates of *Mal^−/−^* mice revealed no changes in the levels of dinitrophenylhydrazone derivatives of protein carbonyls compared to wild-type control mice irrespective of the absence or presence of cigarette smoke exposure (Figure 5A). Consistent with the notion that Mal deficiency in the lungs does not alter oxidative responses, there was no significant alteration in the gene expression of *Nox3* in the lungs of *Mal^−/−^* mice compared to wild-type control mice (Figure 5B). Interestingly, however, emphysema-free *Mal^−/−^* mouse lungs showed an elevated level of apoptosis at 6 months of age (Figure 5C), comparable to that observed in the lungs of emphysematous *Tlr4^−/−^* mice (Figure 3D). We have also examined which apoptotic pathway (extrinsic versus intrinsic) is associated with the elevated apoptosis in the *Mal^−/−^* mice by performing immune-staining in the lungs of mice for active-caspase 8 and active-caspase 9. While we could not detect any caspase-9 staining in the lungs of *Mal^−/−^* (or wild-type) mice, caspase 8 was significantly increased in *Mal^−/−^* mice (Figure 5D). Therefore, these data suggest that Mal selectively promotes the pulmonary anti-apoptotic, but not oxidant suppressive, activities of TLR4, both of which appear to be essential for TLR4 signaling to preserve the normal architecture of the lung.

## Discussion

Historically, Mal has been assigned an essential role in the context of facilitating inflammatory signaling responses from immune cells downstream of TLRs, namely TLR2 and TLR4 [12]. In this paper, we now provide definitive genetic proof that Mal is not required for the function of TLR4 in protecting against the onset of emphysema. Since it has also been shown that MyD88 (like TLR4) is required to prevent the onset of emphysema [3], these observations reveal a differential requirement for Mal and MyD88 in maintaining normal lung architecture via TLR4 signaling. This finding is analogous to the recently reported non-essential and essential roles for Mal and MyD88, respectively, in a TLR2-driven spontaneous gastric tumour mouse model [36]. In addition, both the onset of emphysema in *Tlr4* ^−/−^ mice and the TLR2-driven gastric tumour mouse model occurs independent of hematopoietic-derived immune cells [3], [36], which suggests the essential role for Mal by TLR2 and TLR4 may be more restricted to immune cells (e.g. macrophages) rather than non-immune (e.g. lung endothelial, gastric epithelial) cells.

To further explore the requirement for Mal in lung pathology, we assessed its role in two cellular mechanisms, apoptosis and oxidative stress, which are associated with emphysema in *Tlr4* ^−/−^ (and *MyD88* ^−/−^) mice. Surprisingly, unlike the dual role of TLR4 and MyD88 in protecting against apoptosis and oxidative stress in the lung, our data indicated a specific role for Mal in protecting only against lung alveolar cell death. This observation is supported by the finding that MyD88 complexed with PI3K is recruited to TLR4 via Mal for activation [37], an event which is essential to activate the PI3K/Akt cell survival pathway. In contrast, TLR4 has been reported to mediate pro-apoptotic signaling in airway epithelial cells of COPD smokers [38] in a different setting (smoke related) to the spontaneous emphysema observed in *Tlr4* ^−/−^ mice [3]. While the above studies do not identify the signaling pathways downstream of TLR4 that either promote or suppress the onset of apoptosis in the lung, it is conceivable that multiple TLR signaling pathways are involved (e.g. NF-κB, ERK MAPK, PI3K/Akt), as has recently been reported for TLR-driven anti-apoptotic responses during gastric tumourigenesis [39].

Our study also revealed that unlike TLR4 and MyD88 [3], Mal is not required to balance oxidative stress levels to maintain lung homeostasis. In this respect, and in contrast to our findings, several studies have indicated a role for Mal in mediating oxidative stress in inflammatory disease conditions [40]–[42], including sepsis-associated acute lung injury [43]. Similarly, a detrimental role for MyD88 by activating oxidative stress has been implicated in acute lung injury [44]–[46]. As per our earlier discussion, we speculate that such differences in the role of Mal in emphysema versus inflammatory disorders are most likely attributed to differences in the disease-associated cell type(s), as well as the nature of the upstream TLR stimuli leading to Mal activation. Therefore, it is possible that TLR4/MyD88 and TLR4/Mal both activate the classical NF-κB pathway which eventually leads to lung injury [44], while the pulmonary TLR4/MyD88 axis specifically via the Nox system regulates oxidative stress levels to maintain lung homeostasis [3].

Another key finding of our study was the *in vivo* evidence that TLR2 (unlike TLR4) is not required to maintain the structural integrity of the lung. The role of TLR2 in the disease pathogenesis of COPD patients is controversial. For instance, it has been shown that some TLR2 polymorphisms augment the severity of COPD as a result of a decline in lung function and increased inflammatory cell numbers [47]. The expression levels of TLR2 in stable COPD patients and healthy smokers decreased significantly, and the down-regulation of TLR2 is associated with reduced lung function parameters [48]. On the other hand, the expression of TLR2 is up-regulated in peripheral blood monocytes harvested from COPD patients, either when clinically stable or during an exacerbation of the disease, as compared to never smokers or smokers with normal lung function. In addition, no significant association of TLR2 polymorphisms with either the onset or the course of COPD has been reported [49], and no alterations in *TLR2* gene expression has been observed in the nasal epithelium of smokers compared with non-smoking controls [50].

We also point out that it is now emerging, albeit from limited *in vitro* studies involving macrophages, that TLR2-induced activation of NF-κB and MAPK signaling is mainly reliant on MyD88, while Mal is primarily required for the TLR2-dependent activation of these signaling cascades in response to low ligand concentrations [14], [15], [51], [52]. A differential requirement for Mal in TLR2 signaling also extends to activation of the PI3K/Akt pathway, where signaling through TLR2/6, but not TLR1/2 heterodimers, requires Mal [14], [15], [51], [52]. We therefore propose that the reason *Tlr4* ^−/−^ mice develop emphysema while *Tlr2* ^−/−^ mice do not may be attributable, at least in part, to qualitative and quantitative differences in the TLR2 and TLR4 ligands in the lung (currently unknown) which are required to activate TLR4 to protect against the onset of emphysema.

In conclusion, our current study supports a differential requirement for Mal and MyD88 in TLR4-mediated emphysema, whereby Mal selectively promotes the pulmonary anti-apoptotic, but not oxidant suppressive, activities of TLR4, both of which appear to be essential for TLR4 signaling to preserve the normal architecture of the lung. Therefore, elucidating the biological consequences of TLR4/MyD88 and TLR4/Mal pulmonary signaling may provide novel therapeutic strategies to protect the lung from oxidant induced injury (re-perfusion injury, ozone and other pollutant) without compromising immunity and mucosal host defence. It is also worth noting that although *Mal* ^−/−^ mice have no evidence of emphysema, the elevated spontaneous apoptosis observed in Mal-deficient mouse lungs suggests that Mal may be a critical determinant in emphysema disease severity in response to bacterial infections which can cause severe disease exacerbation as seen in COPD/emphysema patients. Indeed, Mal is the most polymorphic of the TLR signaling adaptors, and several studies have indicated that single nucleotide polymorphisms in the gene for Mal (*TIRAP*) are associated with increased susceptibility to bacterial infections, including those (e.g. Haemophilus influenzae) related to lung disease [53], [54] Accordingly, it is conceivable that increased apoptosis caused by Mal deficiency in the lungs may predispose to emphysematous changes upon exposure to an additional pathological insult, such as bacterial infection with lung pathogens, which is worthy of future investigation. Finally, this study also provides first *in vivo* evidence for the dispensable role of TLR2, unlike its related TLR family member TLR4, in preventing emphysematous changes within the lung. It will now be of great interest to determine the downstream molecular pathways associated with apoptosis and oxidative stress in maintaining lung homeostasis in TLR4-driven innate immunity, as well as the identity of TLR4-specific ligands involved in these cellular processes.

## Supporting Information

Table S1 (DOC)
