# Supplementary material for: Non-Essential Role for TLR2 and Its Signaling Adaptor Mal/TIRAP in Preserving Normal Lung Architecture in Mice
Source: PLoS One. 2013 Oct 29;8(10):e78095. doi: 10.1371/journal.pone.0078095 (PMC3812132; doi:10.1371/journal.pone.0078095)
Supplement: Table S1 — (DOC) [file pone.0078095.s001.doc]

# **Table S1**

# **Comparative stereological analyses of lungs from wild-type (+/+) and Mal^-/-^ mutant mice.**

|  | ***+/+*** | ***Mal^-/-^*** |
| --- | --- | --- |
| **Vv (par/lung) (%)** | 84.4 (±1.0) | 84.2 (±2.1) |
| **Vv (airsp/par) (%)** | 66.9 (±1.4) | 69.8 (±1.2) |
| **V (airsp/lung) (cm^3^)** | 57.1 (±3.2) | 45.8 (±1.1) |
| **Vv (sep/par) (%)** | 23.9 (±1.1) | 25.6 (±0.6) |
| **V (sep/lung) (cm^3^)** | 19.6 (±1.9) | 17.6 (±0.8) |
| **Sv (sep/par) (1/cm)** | 639 (±36) | 632 (±3.2) |
| **S (sep/lung) (cm^2^)** | 536 (±49) | 523 (±6.7) |

Data are expressed as the mean ± SEM. n = at least 5 mice per genotype. Vv = volume fraction; par = parenchyma; air = air space; sep =septal tissue; Sv = surface density; S = surface area.
